# Supplementary material for: Comparing COVID-19 Vaccination Outcomes with Parental Values, Beliefs, Attitudes, and Hesitancy Status, 2021–2022
Source: Vaccines (Basel). 2022 Sep 28;10(10):1632. doi: 10.3390/vaccines10101632 (PMC9611997; doi:10.3390/vaccines10101632)
Supplement: Supplementary file 1 [file vaccines-10-01632-s001.zip › vaccines-1920962-supplementary.pdf]

Supplementary Table S1 (S1)

**Supplemental Table S1. Children's Hospital of Philadelphia Outpatient Care Network Patient Demographics, 2022.**				
**CHOP Site Type**	**Urban Academic N (%)**	**Urban Non-Academic N (%)**	**Suburban N (%)**	**Total N (%)**
**Patient Characteristics**				
**Gender**				
Female	29,066 (49%)	15,234 (49%)	94,986 (49%)	139,286 (49%)
Male	29,790 (51%)	15,893 (51%)	99,002 (51%)	144,685 (51%)
Unknown	0 (0%)	0 (0%)	3 (0%)	3 (0%)
**Race**				
White or Caucasian	5462 (9%)	12,100 (39%)	125,915 (65%)	143,477 (51%)
Black or African American	42,416 (72%)	13,487 (43%)	22,350 (12%)	78,253 (28%)
Asian or Pacific Islander	2732 (5%)	1277 (4%)	9941 (5%)	13,950 (5%)
Multiple	1355 (2%)	1198 (4%)	5927 (3%)	8480 (3%)
Other or Unknown	6,891 (12%)	3065 (10%)	29,858 (15%)	39,814 (14%)
**Ethnicity**				
Hispanic or Latino	5092 (9%)	2268 (7%)	16,273 (8%)	23,633 (8%)
Non-Hispanic or Latino	53,591 (91%)	28,516 (92%)	176,378 (91%)	258,485 (91%)
Unknown	173 (0%)	343 (1%)	1,340 (1%)	1856 (1%)
**Patient Age**				
< 1 year	9979 (17%)	5663 (18%)	28,478 (15%)	44,120 (16%)
1-5 years	16,021 (27%)	8297 (27%)	49,147 (25%)	73,465 (26%)
6-8 years	9048 (15%)	4125 (13%)	29,053 (15%)	42,226 (15%)
9-12 years	11,560 (20%)	5538 (18%)	39,053 (20%)	56,151 (20%)
13-17 years	10,761 (18%)	6218 (20%)	42,219 (22%)	59,198 (21%)
18-19 years	1487 (3%)	1286 (4%)	6041 (3%)	8814 (3%)
**Payer**				
Medicaid	43,498 (74%)	8648 (28%)	42,268 (22%)	94,414 (33%)
Commercial or Other	15,296 (26%)	22,439 (72%)	151,430 (78%)	189,165 (67%)
Unknown	62 (0%)	40 (0%)	293 (0%)	395 (0%)

Supplementary Table S2 (S2) Survey 1.

PART 1: Demographics
1. First Name
2. Last Name
3. Email Address
4. DOB
5. What sex were you assigned at birth, on your original birth certificate?
a. Male
b. Female
c. Intersex
d. Prefer not to answer
6. How do you currently identify?
a. Man
b. Woman
c. Nonbinary
d. Prefer not to answer
e. I identify my gender as __________________ *(please specify)*
7. Which of the following best describes you?
a. Heterosexual or straight
b. Gay or lesbian
c. Bisexual
d. Prefer not to answer
e. Different identity ____________ *(please specify)*
8. Are you of Hispanic, Latino/a/x, or Spanish origin?
a. No, not of Hispanic, Latino/a/x, or Spanish origin
b. Yes, Mexican, Mexican American, Chicano
c. Yes, Puerto Rican
d. Yes, Cuban
e. Yes, another Hispanic, Latino/a/x, or Spanish origin _________________ *(please specify)*
9. What is your race? (Choose all that apply)
a. White or Caucasian
b. Black or African-American
c. American Indian or Alaska Native
d. Chinese
e. Filipino
f. Vietnamese
g. Korean
h. Japanese
i. Asian Indian
j. Other Asian: _______________________ (please specify)
k. Native Hawaiian
l. Samoan
m. Guamanian or Chamorro
n. Other Pacific Islander: ________________________(please specify)
o. Some other race: ______________________________(please specify)
10. What is your household income?
a. Less than $25,000/year
b. $25,000 - $49,999/year
c. $50,000 - $74,999/year
d. $75,000 - $99,999/year
e. $100,000 - $149,999/year
f. Over $150,000/year
11. What is the highest degree or level of school you have COMPLETED (Select one. If currently enrolled, mark the previous grade or highest degree received)
a. No schooling completed
b. Regular high school diploma
c. GED or alternative credential
d. Some college credit
e. Associates degree (i.e. AA, AS)
f. Bachelor’s degree (i.e. BA, BS)

g. Master's degree (i.e. MA, MS, MSW, MBA)
h. Profession degree beyond bachelor's (i.e. MD, DOS, JD)
i. Doctorate degree (i.e. PhD, EdD)

Child Demographics

12. How many children do you have age 18 and under?
a. 1
b. 2
c. 3
d. 4
e. 5
f. 6
13. What are the ages of each child under 19? [BRANCH: Answer for each child separately and program survey in this way]
a. Under 1
b. 1
c. 2
d. 3
e. 4
f. 5
g. 6
h. 7
i. 8
j. 9
k. 10
l. 11
m. 12
n. 13
o. 14
p. 15
q. 16
r. 17
s. 18

14. What is the sex of each child?
a. Female
b. Male
c. Intersex
d. Prefer not to answer

PART 2: Routine Vaccine Acceptance (Q9-16)

The following questions are about routine childhood vaccinations that are recommended for children such as Hepatitis A, Hepatitis B, Measles, Mumps, and Rubella (MMR).

15. Are you the primary decision-maker of vaccines for your child(ren)?
a. Yes, I am the primary decision-maker
b. Decisions are made jointly
c. Another parent/guardian is the primary decision-maker
16. Have you personally received a seasonal flu vaccine this past season 2020-2021?
a. Yes
b. No
c. Not sure

Next we're going to ask you a few questions about your child's or children's vaccinations. If you have more than one child, we will ask you questions about each child.

17. Did your child under 19 receive a seasonal flu vaccine this past season 2020-2021? [Answer for each child separately and program survey in this way]
a. Yes
b. No
c. Not sure
18. Is your child under 19 currently a Children's Hospital of Philadelphia patient?
a. Yes, currently → [ask for First name and last name, DOB]
b. No, but has been in the past → [ask for First name and last name, DOB]
c. No, never has been a patient → [ask for First name and last name, DOB to double check]

19. Has your child received all the recommended vaccines for their age?
a. Yes
b. No
c. Don't know
20. If no, which ones have they not received for their age. Please check all that apply. [BRANCH each child]
a. Hepatitis B
b. Rotavirus
c. Diphtheria/Tetanus/Pertussis
d. Haemophilus influenzae type b
e. Pneumococcal
f. Polio
g. Measles, Mumps, Rubella (MMR)
h. Varicella (chickenpox)
i. Hepatitis A
j. Human papillomavirus (HPV)
k. Meningococcal
l. Don't know
21. (Adapted from source Hemlkamp et al, 2021) Please indicate whether you disagree or agree with the following statements:

	Strongly agree	Agree	Disagree	Strongly disagree
Vaccines are important for my child's health				
Getting a vaccine is a good way to protect my child from developing vaccine-preventable diseases.				
Childhood vaccines are effective.				
Having my child get all of the recommended vaccines is important for the health of others in my community.				
All childhood vaccines offered by my child's health care provider are beneficial.				
I do what my child's health care provider recommends about vaccines.				
Some vaccines have not been around long enough to ensure that they are safe.				
I am concerned about serious side effects of some vaccines.				
I think some vaccines might cause lasting health problems for my child.				

PART 3: COVID-19: Thinking/Feeling, Motivation, Social Processes and Return to school

The next set of questions are about the COVID-19 vaccine.

22. Have you personally received a COVID-19 vaccine?
a. Yes, I am fully vaccinated
b. Yes, one dose of a two-dose series
c. No
23. (KFF Attend School 2021) During the 2021-2022 school year, is your youngest child attending school…
a. All in-person
b. Mostly in-person

c. A mix of in person and online
d. Mostly online
e. All online
24. (PERCEIVED RISK) How concerned are you about your youngest child getting COVID-19 at school?
a. Not at all concerned
b. A little concerned
c. Moderately concerned
d. Very concerned
25. (VACCINE SAFETY) How safe do you think the COVID-19 vaccine is or will be for your youngest child when he or she is eligible?
a. Not safe at all
b. A little safe
c. Moderately safe
d. Very safe
26. (COVID-19 MORBIDITY/MORTALITY IN SOCIAL NETWORK) Do you personally know anyone in your family, group of friends, or community networks who became seriously ill or died as a result of COVID-19?
a. Yes
b. No
c. Not Sure
27. Has your child who is age-eligible received a COVID-19 vaccine? [Program for each child]
a. Yes, already received and fully vaccinated
b. Yes, one dose of a two-dose series
c. No
28. BRANCH IF NO: If Q-above is No: (INTENTION) Do you intend to get your age-eligible child a COVID-19 vaccine?
a. Yes – when it is fully licensed by the FDA for children of all ages
b. Yes - regardless of the fact the vaccine is not fully FDA licensed for children of all ages
c. Yes - but not sure when
d. Not sure, I'm still deciding
e. No
29. **Branch if Q26 is NO** (KFF Reason for not vaccinating children 12+) What are the main reasons your child age-eligible child has not gotten a COVID-19 vaccine? Select all that apply
a. Need more information/tests/research
b. Side effects/reactions
c. Child doesn't want it/their choice
d. Not worried about COVID-19/Don't think vaccine is necessary
e. Not allowed by parents
f. Don't trust the vaccine
g. Worried about safety
h. Scheduling/have not been able to go
i. Not eligible/vaccine not available
j. Fear/afraid of getting it
k. Want to wait and see
l. Already had COVID-19/been exposed
m. Medical condition/existing health problems
n. Concerns about long term effects
o. Don't like needles/shots
p. Heart problems
q. Infertility concerns
r. Other: please specify________
30. BRANCH Under12: If the child is not eligible for the COVID-19 vaccine due to his/her age: Under 12 Intention (INTENTION) When a COVID-19 vaccine is available for your child (under age 12 years) will you get your child vaccinated
a. Yes, will get it as soon as possible
b. Yes, but plan to wait to get it
c. Not sure, I need more information
d. Definitely not
31. (ACCESS PREFERENCE) Where would you prefer to get your child vaccinated (select top choice)?
a. At school
b. At pediatrician/physician's office

c. Health department clinic
d. Other clinic or health center
e. Hospital
f. Pharmacy or drug store (e.g., Walgreens or CVS)
g. Pop-up clinic in my community (e.g. a health fair or community social event)
h. Other, please specify______________
i. No preference
j. Not sure

32. (COVID-19 MOTIVATOR BELIEFS) Which of the following statements do you agree with the most (select top choice): Getting my child vaccinated will…
a. Protect my child
b. Protect family/friends
c. Protect others in school/community
d. Allow my child(ren) to resume social activities and sports
e. Allow my child(ren) to resume travel
f. All of the above
g. Other, please specify ___________

33. BRANCH 12yo+ (SOCIAL NORM 12+) How many of your child's close friends do you think are fully vaccinated with the COVID-19 vaccine?
a. None
b. A few
c. About half
d. Most
e. All
f. Don't know

34. BRANCH 12yo+ (SCHOOL NORM 12+) How many of the other eligible students at your school and their eligible family members do you think are fully vaccinated with the COVID-19 vaccine?
a. None
b. A few
c. About half
d. Most
e. All
f. Don't know

35. BRANCH 12yo+ (Adapted from KFF: School vaccine guidance question) Thinking about your 12 years old or older child, has your child's school…? (Select all that apply)
a. Provided you with information about how to get a COVID-19 vaccine for your child
b. Encouraged parents to get their children vaccinated
c. Said they will require students to be vaccinated for COVID-19 in order to return to school in-person
d. Asked about your child's COVID-19 vaccination status
e. No there is no guidance from the school about vaccinating children
f. Not sure

36. (KFF School Mask Mandate) <u>GRID</u> Do you think your children's school should implement these specific COVID-19 safety measures: (1) Require students to be vaccinated for COVID-19 (once FDA authorized for all school-age children) as they do for most other diseases like measles, (2) Require unvaccinated students and staff to wear masks, (Provide voluntary, free weekly COVID-19 testing of children at schools)
a. Yes
b. No
c. Don't know

37. (SOCIAL NORM/INTENT) <u>GRID</u> If you were personally vaccinated how likely would you and your child be to: (1) Wear a mask while indoors, (2) Stand 6 feet away from others, (3) Avoid public transit, (4) Avoid traveling out of state, (5) Avoid indoor dining, (6) Avoid indoor gatherings
a. Not at all likely
b. Somewhat likely
c. Very likely

PART 4: COVID-19 Access questions

38. (CDC Access – for unvaccinated children 12+) How easy do you think it will be to get a COVID-19 vaccine for your child? Would you say…
a. Very easy
b. Somewhat easy

c. Somewhat difficult
d. Very difficult
e. Not sure

39. (CDC Access – for vaccinated children ages 12+) How easy was it to get a COVID-19 vaccine for your child? Would you say…

a. Very easy
b. Somewhat easy
c. Somewhat difficult
d. Very difficult
e. Not sure

40. (CDC Access question – for unvaccinated children of 12+ skip if answered "very easy" to question 38.) What makes it difficult for you to get a COVID-19 vaccine for your child? Select all that apply

a. I'm too busy to go with my child.
b. My family members don't want me to vaccinate my child.
c. It's too far away.
d. I don't know where to go to get my child vaccinated.
e. My child isn't eligible to get a COVID-19 vaccine.
f. My child has a medical reason that makes him/her ineligible to get vaccinated (e.g., He/she has had a severe allergy to vaccines in the past).
g. I don't have transportation.
h. The hours of operation are inconvenient.
i. The waiting time is too long.
j. It is difficult to find or make an appointment.
k. It was difficult to arrange for childcare for my other children.
l. I don't have time off work or school to take my child.
m. Other
n. Not sure

PART 5: Comments. Please provide any comments you wish in this field here.

Supplementary Table S3 (S3) Survey 2.

PART 1: PARENT DEMOGRAPHICS AND COVID-19 VACCINE STATUS

Parent Demographics and COVID-19 Vaccine Status

1. Parent/Guardian First Name
2. Parent/Guardian Last Name
3. Parent Email Address
4. Parent DOB
5. Since we last asked, have you personally received a COVID-19 vaccine?

a. I was fully vaccinated before the first survey and have not received a booster
b. I was fully vaccinated before the first survey and since have received a booster
c. Yes, I have been fully vaccinated since the first survey
d. Yes, one dose of a two-dose series since the first survey
e. Yes, and I also received a booster dose since the first survey
f. No

Parent Perceptions and Behavior

The following questions are about vaccines in general. Please indicate your level of agreement with the following statements from Strongly agree to Strongly Disagree : 1 = Strongly Agree, 2= Agree, 3= Somewhat Agree 4= Somewhat Disagree, 5 = Disagree, 6=Strong Disagree

6. I feel safe after being vaccinated.
7. I can rely on vaccines to stop serious infectious diseases.
8. I feel protected after getting vaccinated.
9. Although most vaccines appear to be safe, there may be problems we have not yet discovered.
10. Vaccines can cause unforeseen problems in children.
11. I worry about the unknown effects of vaccines in the future.
12. Vaccines make a lot of money for pharmaceutical companies, but do not do much for regular people.
13. Authorities promote vaccination for financial gain, not for people's health.
14. Vaccination programs are a big con.
15. Natural immunity lasts longer than a vaccination.
16. Natural exposure to viruses and germs gives the safest protection.
17. Being exposed to diseases naturally is safer for the immune system than being exposed through vaccination.
18. Have YOU received an influenza (Flu) shot since August of 2021 (this influenza season)?

a. Yes
b. No, but I intend to
c. No

19. Have you ever refused or chosen not to get a recommended vaccine for you or someone you are responsible for (e.g., your child)?

a. Yes
b. No

PART 2: CHILD DEMOGRAPHICS AND COVID-19 VACCINE STATUS

20. How many children do you have age 18 and under?

a. 1
b. 2
c. 3
d. 4
e. 5
f. 6

21. Is your child under age 19 currently a Children's Hospital of Philadelphia patient [Answer for each child separately]

a. Yes, currently → [ask for First name and last name, DOB]
b. No, but has been in the past → [ask for First name and last name, DOB]
c. No, never has been a patient → [ask for First name and last name, DOB to double check]

22. What is the age of child? [Answer for each child separately]

a. Under 1-18

23. Has [CHILD NAME] received an influenza shot since August of 2021 (this season)? [Program for each child]

a. Yes
b. No, but I intend to vaccinate them
c. No

Child COVID-19 Vaccine Status and Intentions

24. Since the time you filled out the first survey Has [CHILD NAME] received a COVID-19 vaccine? [Program child name for each age-eligible child, 5 years and older]
a. Yes, already fully vaccinated at the time I completed the first survey
b. Yes, completed the second dose between filling out the first survey and this current one.
c. Yes, fully vaccinated after the completion of the first survey
d. Yes, received one dose of a two-dose series since the completion of the first survey
e. No
25. BRANCH IF NO: If Q-above is No: (INTENTION) Do you intend to get [CHILD NAME] a COVID-19 vaccine?
a. Yes – we have an appointment to get the vaccine
b. Yes – but we do not have an appointment yet
c. Yes - but not sure when
d. Not sure, I'm still deciding
e. Highly unlikely in the near future
f. No

PART 3: COVID-19: THINKING/FEELING, MOTIVATION, SOCIAL PROCESSES, AND RETURN TO SCHOOL

The next set of questions are about the COVID-19 vaccine specifically. BRANCH 5yo+ Prompt if multiple children ages 5+. For the following questions, please think about [CHILD NAME*] and the COVID-19 vaccine.

26. BRANCH 5yo+ Branch if Q26 is NO (KFF Reason for not vaccinating children 5+) What are the main reasons [CHILD NAME*] has not gotten a COVID-19 vaccine? Select all that apply
a. Need more information/tests/research
b. Side effects/reactions
c. Child doesn't want it/their choice
d. Not worried about COVID-19/Don't think vaccine is necessary
e. Waiting for it to be full approval by the FDA instead of Emergency Use Authorization
f. Waiting for the vaccine to be available to children of all ages from six months and older
g. Not allowed by parents
h. Other family members don't want child to get it
i. Don't trust the vaccine
j. Worried about safety
k. Scheduling/have not been able to go
l. Not eligible/vaccine not available
m. Fear/afraid of getting it
n. Want to wait and see
o. Already had COVID-19/been exposed
p. Medical condition/existing health problems
q. Concerns about long term effects
r. Don't like needles/shots
s. Heart problems
t. Infertility concerns
u. Other: please specify_______
27. BRANCH 5yo+ (VACCINE SAFETY) How safe do you now think the COVID-19 vaccine is for [CHILD NAME*]?
a. Not safe at all
b. A little safe
c. Moderately safe
d. Very safe
28. BRANCH 5yo+ (Provider recommendation 5+) Has [CHILD NAME*]'s pediatrician or health care provider recommended that he or she receive the COVID-19 vaccine?
a. Yes
b. No
c. Not sure
29. BRANCH 5yo+ (Friend/Family members) Have your close family members or friends recommended to you that [CHILD NAME*] receive the COVID-19 vaccine?
a. Yes
b. No
c. Not sure
30. BRANCH 5yo+ (SOCIAL NORM 5+) How many of [CHILD NAME*]'s close friends do you think are fully vaccinated with the COVID-19 vaccine?

a. None
b. A few
c. About half
d. Most
e. All
f. Don't know

31. BRANCH 5yo+ (SCHOOL NORM 5+) How many of the other eligible students at [CHILD NAME*]'s school and their eligible family members do you think are fully vaccinated with the COVID-19 vaccine?

a. None
b. A few
c. About half
d. Most
e. All
f. Don't know

32. BRANCH 5yo+ (Adapted from KFF: School vaccine guidance question) Has [CHILD NAME]'s school…? (Select all that apply)

a. Provided you with information about how to get a COVID-19 vaccine for your child
b. Encouraged parents to get their children vaccinated
c. Said they will require students to be vaccinated for COVID-19 in order to return to school in-person
d. Asked about your child's COVID-19 vaccination status
e. There is no guidance from the school about vaccinating children
f. Not sure

33. BRANCH 5yo+ (KFF School Mask Mandate) Has [CHILD NAME*]'s school implemented any of these specific COVID-19 safety measures: Select all that apply:

a. Required students to be vaccinated for COVID-19
b. Required unvaccinated students and staff to wear masks
c. Provide voluntary, free weekly COVID-19 testing of children at schools
d. None

34. (SOCIAL NORM/INTENT) <u>GRID</u> How often do you: (1) Wear a mask while indoors, (2) Stand 6 feet away from others, (3) Avoid public transit, (4) Avoid traveling out of state, (5) Avoid indoor dining, (6) Avoid indoor gatherings

a. Almost always
b. Most of the time
c. About half of the time
d. Some of the time
e. Almost never
f. Never

35. BRANCH 5yo+ SOCIAL NORM/INTENT) <u>GRID</u> How often does your child(ren): (1) Wear a mask while indoors, (2) Stand 6 feet away from others, (3) Avoid public transit, (4) Avoid traveling out of state, (5) Avoid indoor dining, (6) Avoid indoor gatherings

a. Almost always
b. Most of the time
c. About half of the time
d. Some of the time
e. Almost never
f. Never

PART 4: COVID-19 ACCESS QUESTIONS

36. (CDC Access – for unvaccinated children 5+) How easy do you think it will be to get a COVID-19 vaccine for your child? Would you say…

a. Very easy
b. Somewhat easy
c. Somewhat difficult
d. Very difficult
e. Not sure

37. (CDC Access – for vaccinated children ages 5+) How easy was it to get a COVID-19 vaccine for your child? Would you say…

a. Very easy
b. Somewhat easy

c. Somewhat difficult
d. Very difficult
e. Not sure
38. (CDC Access question – for unvaccinated children of 5+ skip if answered "very easy" to question 38.) What makes it difficult for you to get a COVID-19 vaccine for your child? Select all that apply
a. I'm too busy to go with my child.
b. My family members don't want me to vaccinate my child.
c. Vaccination sites are too far away.
d. I don't know where to go to get my child vaccinated.
e. My child isn't eligible to get a COVID-19 vaccine.
f. My child has a medical reason that makes him/her ineligible to get vaccinated (e.g., He/she has had a severe allergy to vaccines in the past).
g. I don't have transportation.
h. The hours of operation are inconvenient.
i. The waiting time is too long.
j. It is difficult to find or make an appointment.
k. It was difficult to arrange for childcare for my other children.
l. I don't have time off work or school to take my child.
m. Other
n. Not sure

PART 5: CORONAVIRUS ANXIETY SCALE
Prompt: We will now ask some questions about your personal experience with COVID-19.
39. Have you or any close family members or friends been infected with COVID-19? [Select all that apply]
a. Yes, me
b. Yes, a close family member or friend
c. No
40. To what extent do you agree that the U.S. government will be able to protect citizens and manage COVID-19 as it continues to change?
a. Completely agree
b. Agree
c. Somewhat agree
d. Somewhat disagree
e. Disagree
f. Completely disagree

How often have you experienced the following activities over <u>the last 2 weeks?</u> [Grid responses for items 40-44, Response Range: Not at all (0); Rare, less than a day or 2 (1); Several days (2); More than 7 days (3); Nearly every day over the last 2 weeks (4)]
41. I felt dizzy, lightheaded, or faint when I read or listened to news about coronavirus.
42. I had trouble falling or staying asleep because I was thinking about the coronavirus.
43. I felt paralyzed or frozen when I thought about or was exposed to information about the coronavirus.
44. I lost interest in eating when I thought about or was exposed to information about the coronavirus.
45. I felt nauseous or had stomach problems when I thought about or was exposed to information about the coronavirus.

PART 6: SOCIAL MEDIA USE
The final questions in the survey are about your general social media use.
46. Do you have any of the following social media accounts? Select all that apply.
a. Facebook
b. Pinterest
c. Instagram
d. TikTok
e. Twitter
f. Telegram
g. Other: Write in
h. None, I don't use social media

Media and Technology Usage and Attitudes Scale (MTUAS) General Social Media Usage Subscale
Use 10-point frequency scale in a grid for items 46-54: Never (1), Once a month (2), Several times a month (3), Once a week (4), Several times a week (5), Once a day (6), Several times a day (7), Once an hour (8), Several times an hour (9), All the time (10)]
Prompt: For the following questions, please think about the social media site you use most often if you use more than one. How often do you do the following on that social media site?

47. Check your social media page from your computer
48. Check your social media page from your phone
49. Check your social media at work or school
50. Post updates
51. Post photos
52. Browse profiles and photos
53. Read postings and/or watch videos
54. Comment on postings, videos, updates, photos etc.
55. Like a posting, video, update, photo etc.

PART 6: COMMENTS. Please provide any comments you wish in this field here.
